# Environmental stressors, complex interactions and marine benthic communities’ responses

**DOI:** 10.1038/s41598-021-83533-1

**Published:** 2021-02-18

**Authors:** Charlotte Carrier-Belleau, David Drolet, Christopher W. McKindsey, Philippe Archambault

**Affiliations:** 1 Québec-Océan, Department of Biology, Université Laval, 1045, av. de la Médecine, Québec, G1V 0A6 Canada; 2 Maurice Lamontagne Institute, Fisheries and Oceans Canada, 850 route de la Mer, Mont-Joli, G5H 3Z4 Canada

**Keywords:** Climate-change ecology, Ecophysiology, Marine biology, Environmental impact

## Abstract

The increasing number and diversity of anthropogenic stressors in marine habitats have multiple negative impacts on biological systems, biodiversity and ecosystem functions. Methods to assess cumulative effects include experimental manipulations, which may identify non-linear responses (i.e. synergies, antagonisms). However, experiments designed to test these ideas are uncommon, generally focusing on single biological responses. We conducted a manipulative experiment to investigate the isolated and combined effects of warming (+ 6 °C), salinity variation (freshwater pulses or presses), and nutrient enrichment (natural or enriched) following one and three month’s exposure, on responses measured at multiple levels of biological complexity in a simple bivalve assemblage. More specifically, we determined effects on bivalve mortality, growth, shell mineralization, and energy content, as well as microphytobenthos biomass. Salinity variation and nutrient enrichment, individually and combined, caused strong impacts on some of the measured variables and their effect varied through time. In contrast, warming had no effect. Our work highlights the prevalence of antagonistic interactions, the importance of examining effects of single and multiple stressors through time, and of considering multiple responses to understand the complexity behind stressor interactions.

## Introduction

Humans have a considerable influence on ecosystems, and scientific evidence of the human footprint on terrestrial, freshwater, and marine ecosystems is now irrefutable^1–3^. Continuous growth of human populations and use of natural systems have increased the number and diversity of anthropogenic environmental stressors^4^—here defined as factors (of natural or anthropogenic origin) perturbing an ecosystem beyond its natural limits of variation^5^. Human activities (e.g. agriculture, fishing, coastal development) together with anthropogenically induced global climate change (e.g. warming) are provoking profound and irreversible changes in coastal marine ecosystems through the emergence of multiple stressors, such as organic pollution, elevated temperature, and salinity changes^6^. Such stressors impact biological processes, alter ecosystem functions, and decrease global and local biodiversity^4,7–9^.

While stressors may occur in isolation, globally 97.7% of the ocean is currently affected by more than one stressor^10^. The spatial and temporal superposition of stressors catalyzes the emergence of complex relationships: i.e. synergistic or antagonistic interactions^7^. Synergistic interactions occur when the combined effect of two or more stressors are greater than the sum of the individual stressors (i.e. additive effect), commonly considered as the null model. In contrast, when the combined effect of multiple stressors leads to a smaller response than that predicted by the null model, the interaction is considered antagonistic^11–14^. When multiple stressors act in opposite directions on a biological response, the interpretation of these interactions becomes even more complex. For instance, if the combined response of two stressors is more positive than the additive expectation, it would be considered a positive synergistic effect. In contrast, if the combined response is more negative than the additive expectation it would be deemed a negative synergistic effect. The same logic applies to antagonistic interactions^14,15^. However, one stressor may dominate another and account for 100% of the biological response measured (i.e. dominance effect). While determining the presence of complex interactions is challenging, it is essential to identify environmental priorities for conservation actions in coastal areas, which are typically affected by greater than 100 two-way interactions between stressors^7,16,17^.

In the context of multiple stressors, identifying synergistic and antagonistic interactions is of particular importance to inform management decisions since they may affect ecosystem responses to interventions^18^. This may be done by informing environmental managers on which stressors to act upon and when and where it is most effective or necessary to intervene^16,17,19,20^. For example, if a single stressor acts synergistically with several others, then action upon this one might generate a disproportionate positive ecosystem response. On the other hand, antagonistic interactions may lead to overall negative effects if all stressors are not addressed together.

However, an important disconnect exists between the investigation of the combined effects of multiple stressors and the implementation of conservation and management practices^7,20^. In this context, a perfect understanding and prediction of the effects of multiple stressors is an unrealistic objective^21^. Identifying generalities about stressors, responses, and interaction types may better inform decision makers in their conservation and legislative actions. To do so, it is essential to design ecologically realistic experiments that consider the impact of local stressors (e.g. nutrient input, overfishing) within the context of global stressors (e.g. climate change)^16^. Global stressors cannot be halted by local actions whereas local stressors may be acted upon effectively and directly manipulated through management actions^16^. For example, reducing a local stressor, such as fishing or nutrient input, may improve recovery rates from uncontrollable stressors, such as climate change^22^.

Many tools exist to understand biological consequences of natural and anthropogenic stressors at physiological^23^, population^24,25^ and ecosystem levels^26,27^. However, effects of stressors on populations may differ from those on individuals, making it crucial to better understand the mechanisms affecting biological organization by looking at the effect of multiple stressors on multiple responses^13,15,28, 29^.

Of the emerging stressors in shallow coastal areas, eutrophication has gained considerable attention over recent decades^30^. Despite this, the effects of nutrient enrichment on benthic communities remain poorly understood, especially in combination with other stressors^31–33^. The influence of salinity changes has also been a central focus of studies on estuarine ecosystem biodiversity, which is commonly reduced along haline gradients from marine to brackish conditions^34,35^. For instance, salinity changes in estuaries may arise from high discharge during freshets, heavy rain events—which are expected to increase in intensity and occurrence due to climate change, wastewater discharges, or upriver flow regulation (e.g. hydropeaking)^36,37^. In addition, global warming represents a major driver of ecosystem change as it threatens habitats, ecological communities, and many natural processes^38,39^. For example, warming is frequently suggested to exacerbate the effects of local stressors, such as nutrient enrichment, which, together, may enhance macroalgal recruitment and triggering blooms in coastal waters^40,41^.

The aim of this study was to determine the short-term effect of multiple stressors alone and in combination on multiple biological responses in two model bivalve species and the type of interactions resulting from stressor combinations using an experimental approach. More specifically, based on the environmental context and parameters measured in an anthropized bay of the Gulf of St. Lawrence estuary (Quebec, Canada)^42^, we concentrated our research effort on determining the impacts of anthropogenically induced nutrient inputs, short-term salinity variations, and elevated temperature. The combined and individual effects of these stressors were evaluated by measuring mortality, growth, shell mineralization, and energy content in the blue mussel *Mytilus* sp. and Baltic clam *Limecola balthica*, as well as chlorophyll *a* in sediments as a measure of microphytobenthic biomass after one- and three-months exposure to these stressors. When stressors interacted, we determined the nature of the effect, discriminating between dominant, additive, synergistic and antagonistic interactions and how it varied after the two exposure periods. We hypothesized that the three stressors would act in isolation (i.e. dominance effect, additive) or interact (i.e. synergistic and antagonistic interaction) depending on the response variable, knowing stressors, such as temperature, nutrient enrichment and salinity variation, have different effects depending on the biological compartment of interest^13,15^.We also hypothesized that the individual and combined effect of the stressors would vary through time, as it has been suggested that the effect of multiple stressors depends on the time scale^43^.

These two bivalve species, like many benthic species, are both sedentary and exposed to stressors in natural ecosystems; they are thus logical indicators and models to assess the effect of stressors^44^. *Mytilus* sp. can survive at considerably low salinities, but growth and biomass increase once the salinity reaches 25 psu^45^, while *L. balthica* can live and reproduce successfully in a broader range of salinities (2–35 psu)^46^. Nutrient enrichment, on the other hand, will logically increase organic matter and may lead to mortality in *Mytilus* sp.^47^ although it has been suggested that *L*. *balthica* may benefit from nutrient enrichment and be used as an indicator of organic pollution^48^. Both species commonly inhabit intertidal areas and are thus accustomed to large temperature fluctuations. For example, *Mytilus* sp. can tolerate temperatures ranging from 3 to 27 °C in the study area, with growth optimum between 10 and 20 °C^45^, while *L. balthica* is associated to cooler temperatures^49^. *L. balthica* may, however, tolerate short exposure to elevated temperatures (37.5 °C), but may experience an energetic lost associated with a sub-optimal metabolic function^49^.

It is hoped that this study will demonstrate the potential complexity of interactions between contemporary multiple stressors on primary and secondary production, thus stressing the difficulty of undertaking logical environmental management actions in the face of such complexity.

## Materials and methods

### Sediment collection, characterization and preparation

We collected sediments in the bay of the Mitis River (48° 38′ N, 68° 08′ W), a tributary of the St. Lawrence estuary (QC, Canada), and transported them to the adjacent Institute Maurice-Lamontagne (Fisheries and Oceans Canada, Mont-Joli, QC, Canada) in June 2018. Sediments were sieved on a 500-μm mesh to remove all macroinfauna while retaining the microphytobenthos. Sediments were then transferred into 80 PVC cylinders used as aquaria (height = 30 cm, width = 10 cm, sediment height = 12 cm) and placed inside the experimental system one week before introducing the bivalves. To characterize initial conditions, Chlorophyll *a*, grain size, and organic matter were measured in the sediment of ten randomly selected aquaria using standard techniques a week prior to the start of the experiment^50,51^. We measured chlorophyll *a* in the first cm of sediments as an indicator of microphytobenthos biomass using a protocol adapted from Riaux-Gobin and Klein^50^. On average, we found 1.0380 µg (SE = 0.0880) of chlorophyll *a* g^−1^ dry sediment. Sediment grain size was determined for the top five cm of sediments using a Laser Diffraction Particle Size Analyzer (LA-950, HORIBA Scientific, Kyoto, Japan) and classified sediment types as follows: mud (< 3.9 μm), silt (3.9 μm < X < 62.5 μm), sand (62.5 μm < X < 2 mm) and gravel (> 2 mm)^52,53^. An average of 54.34% (SE = 0.5866) of sediment particles was sand whereas 38.8% (SE = 0.2171) was mud. Initial percentage organic matter was characterized following Davies^51^ and averaged 3.26% (SE = 0.0009). Chlorophyll *a*, grain size, and organic matter did not differ substantially among analyzed aquaria and any variation was assumed to be random.

### Organism collection, transport and maintenance

Blue mussels (*Mytilus* sp.) (Linnaeus, 1758) and Baltic clams (*Limecola balthica)* (Linnaeus, 1758) were collected by hand in the same bay as the sediment in June 2018. Specimens were transported to the laboratory in water buckets within an hour of collection and were blotted, measured (length, width, height) and weighed. They were then tagged on the right outer shell using bee marking numbers (The Bee Works, Orillia, ON, Canada). Specimens were maintained two months in the laboratory in a tank supplied with a continuous flow of unfiltered seawater (1.500 L min^−1^). After 2 months of acclimation to laboratory conditions, specimens were introduced to the experimental system. We selected juveniles ranging from 2 to 2.5 cm for *Mytilus* sp. and 1 to 1.5 cm for *L. balthica.* Eight individuals of each species were randomly placed together in each aquarium, this reflecting their naturally occurring densities at the sampling site (mean ± SE: 15 m^−2^ ± 0.56 and 14·m^−2^ ± 0.54, respectively), for a total of 640 *per* species. The two species occur together in the St. Lawrence; while *Mytilus* sp. is found attached on rocks or on soft substrates, *L. balthica* is found only in sediments.

### Experimental design, system and protocol

The experiment was performed between August 1^st^ and October 23^rd^, 2018. We assigned each aquarium randomly to one of the 16 experimental treatments following a full-factorial design (n = 5 aquaria *per* treatment) (Table 1, Supplementary Figure S1).

**Table 1:** Mean (± SE) values of experimental parameters measured or calculated in mixing tanks (temperature, salinity) or aquaria (nutrients) over the duration of the experiment for all treatments: phosphorus (P), nitrogen (N), and potassium (K) release (mmol m^−2^d^−1^), temperature (°C), salinity (PSU) and pH during regular conditions and freshwater pulses and presses.

Treatments			Measured experimental parameters								
Temperature	Salinity variation	Nutrient	Nutrient release (mmol m^−2^ day^−1^)			Temperature (°C)	Temperature (pulse/press)	Salinity PSU	Salinity (pulse/press)	pH	pH (pulse/press)
			P	N	K						
Ambient	Pulse	N^−^	–	–	–	7.61 (± 0.04)	7.76 (± 0.04)	27.96 (± 0.05)	20.28 (± 0.06)	8.76 (± 0.01)	8.74 (± 0.01)
Ambient	Pulse	N^+^	39.6928 (± 1.7893)	213.1646 (± 9.6091)	44.9218 (± 2.0250)	7.61 (± 0.04)	7.76 (± 0.04)	27.96 (± 0.05)	20.28 (± 0.06)	8.76 (± 0.01)	8.74 (± 0.01)
Ambient	Press	N^−^	–	–	–	7.62 (± 0.04)	7.69 (± 0.04)	27.91 (± 0.04)	11.57 (± 0.03)	8.74 (± 0.01)	8.76 (± 0.01)
Ambient	Press	N^+^	37.8973 (± 0.9893)	203.5212 (± 5.3128)	42.8895 (± 1.1196)	7.62 (± 0.04)	7.69 (± 0.04)	27.91 (± 0.04)	11.57 (± 0.03)	8.74 (± 0.01)	8.76 (± 0.01)
Warming	Pulse	N^−^	–	–	–	13.50 (± 0.03)	14.06 (± 0.04)	28.14 (± 0.05)	20.52 (± 0.05)	8.73 (± 0.02)	8.74 (± 0.01)
Warming	Pulse	N^+^	38.3835 (± 1.6225)	206.1328 (± 8.7132)	43.4399 (± 1.8362)	13.50 (± 0.03)	14.06 (± 0.04)	28.14 (± 0.05)	20.52 (± 0.05)	8.73 (± 0.02)	8.74 (± 0.01)
Warming	Press	N^−^	–	–	–	13.52 (± 0.03)	13.96 (± 0.04)	27.98 (± 0.05)	11.50 (± 0.03)	8.74 (± 0.01)	8.77 (± 0.01)
Warming	Press	N^+^	39.2434 (± 0.8236)	210.7512 (± 4.4231)	44.4132 (± 0.9321)	13.52 (± 0.03)	13.96 (± 0.04)	27.98 (± 0.05)	11.50 (± 0.03)	8.74 (± 0.01)	8.77 (± 0.01)

Pulse treatments corresponded to periodical moderate drops of salinity six times *per* day: 9:00–9:30, 13:00–13:30, 17:00–17:30, 21:00–21:30, 01:0 0–01:30, 5:00–5:30. The press treatment corresponded to a single intense drop of salinity from 9:00 to 12:00.

The intensities of the three stressors were selected to reflect conditions observed in the St. Lawrence and to stay within, but at the limit of, both species’ tolerances to temperature, salinity, and nutrient enrichment. The temperatures used mimicked average temperatures of surface waters in coastal areas of the Gulf of St. Lawrence and surface anomalies recorded in recent years^54^. We used two stable temperatures for the duration of the experiment: ambient temperature recorded at this time of the year (7.5 °C) and an increase of 6 °C (13.5 °C) that corresponds to anomalies recorded in the surface layers (0–50 m) of different locations in the estuary and Gulf of St. Lawrence (Table 1)^54^. For stress induced by salinity variations, we generated pulses and presses of freshwater instead of using stable salinities as being intertidal, the organisms are used to being exposed to freshwater during low tide and salt-water during high tide. The pulse treatment corresponded to periodic drops of salinity, passing from ambient salinity (i.e. 28 psu) to a salinity of 20 psu for 30 min, six times per day. The press treatment corresponded to a single drop of salinity passing from ambient to a salinity of 12 psu for three hours. These treatments were not measured in situ but selected to represent either small natural or anthropogenic variations in salinity (e.g. continuous wastewater release) *versus* presses linked to extreme climatic events caused by global change (e.g. spring freshet or increased river flow). Here, we a priori considered the press treatment to be more stressful, due to its greater variation in intensity and its longer time of exposure. We used natural levels of nutrients of the sediments or an in situ enrichment technique using controlled-release fertilizer pellets (Osmocotes, Acer NT NPK 17-7-10, Plant-Prod, Brampton, ON, Canada) (Table 1). We used 30 g of pellets in mesh bags and dug them into the sediments, 8 cm from the surface, one week prior to the start of the experiment. We weighed the fertilizer pellets before and at the end of the experiment to characterize the daily N, P, and K release. Nutrient release was judged to not differ over time as fertilizer pellet loss per unit time was roughly equivalent following one and three months of experimental conditions (mean ± SE: 5.30 g ± 0.0898 and 15.87 SE ± 0.1278, respectively). The technique and quantity of nutrients released was similar to those used in other experiments simulating nutrient enrichment and investigating their impact on benthic communities^30,55,56^. For natural conditions, we placed mesh bags filled with small pebbles in the sediments to control for possible effects of mesh bags in aquaria.

The experimental set up was an open system consisting of two header tanks, eight independent tanks to obtain the desired mix of salinity and temperatures (mixing tanks, n = 2 salinity-temperature treatment) and 80 aquaria (experimental units, n = 5 per treatment) (Supplementary Figure S1). The desired temperatures were obtained using titanium coils immersed in the head tanks to warm or cool filtered sea water supplied directly from the St. Lawrence and fresh municipal drinking water. Salinity was controlled by regulating salt- and freshwater inputs to mixing tanks using flow meters and timers to obtain a total flow of 150 L h^−1^, which enabled us to avoid the use of an additional aeration system. We used flexible airline tubing to randomly link each mixing tank to ten aquaria, for a total of eighty experimental aquaria. The experimental units were placed in 80 L water baths to keep the sediments at desired temperatures. Each water bath housed two randomly distributed replicates for each salinity/nutrient treatment at a given temperature for a total of 8 aquaria per water bath (Supplementary Figure S1). The aquaria were exposed to a 12-h photoperiod (7:00 to 19:00) by using 6 2′ × 4′ LED panels (72 W) that were placed 30 cm above the water bath. Temperature, salinity, and pH in the eight mixing tanks were measured daily during freshwater pulses and presses with a multiprobe (Table 1). Salinity and temperature were also recorded in  ten haphazardly selected aquaria during each freshwater pulse and press to ensure experimental conditions did not differ from those in mixing tanks. Oxygen was measured ten times in all aquaria during the experiment and never fell below 90% at the surface of the sediments. Due to unfortunate events, two experimental units were lost after three months of exposure (Warming-Press-N^−^ and Ambient-Press-N^−^).

Organisms were fed twice per day with Shellfish diet 1800 (Reed mariculture, Campbell, CA, USA)—a mix of six marine microalgae: *Isochrysis* strain (CCMP1324), *Pavlova* strain (CCMP459), *Tetraselmis* strains (PLY 429), *Chaeotoceros calcitrans*, *Thalassiosira weissflogii* and *Thalassiosira pseudonana*. 180 mL of Shellfish diet 1800 was diluted in 1.7 L of sea water and 21.25 mL was transferred to each aquarium with a graduated plastic pipette at 10:00 and 16:00. After one- and three-months exposure, half of the aquaria in each water bath (i.e. one of each treatment) were collected and processed as described below.

### Chlorophyll a biomass

Chlorophyll *a* was evaluated as a proxy for microphytobenthos biomass in our experimental units. The upper 1 cm of sediments from each aquarium was sampled with a 10 mL disposable syringe, placed in a 15 mL Falcon tube, and frozen at − 80 °C for subsequent analysis. Pigment concentrations were determined fluorometrically (Turner 10AU, San Jose, CA, USA) following a modified Riaux-Gobin and Klein protocol^50^. The results are expressed as μg g^−1^ dry sediment.

### Mortality and growth

At the end of the experiment, individual bivalves were carefully retrieved from the sediments. Mortality, defined as the percentage of dead individuals for each species, was determined by identifying dead individuals and empty shells. Shell metrics (length, width, height) and mass of each surviving individual were taken and compared to initial sizes to characterize growth (mm). Even though juveniles invest more energy in growth than do adults^57^, thus justifying our focus on juveniles, we did not expect growth, as bivalve growth is significantly reduced by August in the St. Lawrence^58^. Individuals were then dissected and the mass of each shell and tissue measured using a precise scale (blotted weight). Shells were individually placed in parafilm and tissues in Eppendorf tubes and both frozen at − 80 °C until they were evaluated for minerals and energy content, respectively.

### Shell mineralization

Shell mineral concentrations were determined as marine organisms possess calcium carbonate structures that provide protection, support for muscle contraction, and are directly and indirectly involved in various physiological functions (e.g. acid–base balance and osmo-ionic regulation)^59^. In particular, we focused on the major mineral components of the shell: calcium (Ca^2+^), magnesium (Mg^2+^), and strontium (Sr^2+^). In addition, to help interpret the results on potential changes in single cation concentrations, we calculated the ratios between Mg^2+^ and Ca^2+^ and Sr^2+^ and Ca^2+^, which are indicators of change in mineralization status of exoskeletal structures^60–62^. Changes in concentrations of given minerals can provide evidence for potential dissolution of the shell or plastic mineral responses. For example, a lower concentration of magnesium and a lower Mg^2+^–Ca^2+^ ratio suggest the shell may be more tolerant to acidification but more vulnerable to mechanical stresses or predation^63,64^. To control for passive dissolution of the shells under all experimental conditions, three empty shells of each species were weighed (dry weight) at the beginning of the experiment and placed in each aquarium (total of 15 *per* species *per* treatment). Following one or three-months exposure, shells were recovered and weighed to evaluate loss.

Three *Mytilus* sp. and three *L. balthica* from each aquarium were haphazardly selected and their shells cleaned using plastic tools to avoid mineral contamination (total of 15 *per* species *per* treatment)^65^. We then cryo-lyophilized (Freezone 12 Floor Model, Labconco, Kansas City, USA) the shells for 48 h at − 40 °C to remove residual moisture. Each dry shell was then weighed, ground, and 0.056 g (SE = 0.0007) placed in a 15 mL Falcon tube for digestion. For digestion, we added 3 mL of nitric acid (65–70%) and 1 mL of hydrogen peroxide (25–35%). After 24 h, we placed samples for 2 h in a sonication bath at 45 °C to accelerate digestion. Once the samples were completely digested, shell minerals were determined at Geo Labs (Sudbury, ON, Canada) using an inductively coupled plasma (ICP) optical emission spectrometer (Spectro Arcos II ICP-OES, SPECTRO, Kleve, Germany) in axial configuration using the custom analytical method based on the parameters of the current water analysis method on the ICP-AES, IAW-200. Geo Labs proceeded to dilutions of a factor of 50 to have sufficient solution for the analysis and to match the instrument’s tolerance limits. The results are expressed in mmol kg^−1^ dry shell.

### Energy content

The effects of single and combined stressors on tissue energy content was determined using the soft tissues of the whole body of two individuals *per* aquarium (total of 10 *per* species *per* treatment). The tissues were first weighed and dried 24 h at 65 °C (GO1340A-1 Lindberg/Blue M, Thermo fisher Scientific, Waltham, USA). After 24 h, dry samples were weighed and then ground to a pellet. Due to the relatively small size of samples, we added a known quantity of benzoic acid to the pellets to raise energy outputs to the calibration level. The heat emitted by the benzoic acid was then removed when calculating the energy of the tissues. The analysis was carried out with a semi-microbomb calorimeter (6725, Parr Instrument Co., Moline, IL, USA), a precision thermometer (6772, Parr Instrument) and oxygen bomb (1109A, Parr Instrument) following the protocol established by Siddon et al.^66^. We used 1 g benzoic pellets (3416, Parr Instrument Company, Moline, USA) for calibration at the beginning of each measurement interval. To control for the heat potentially emitted by the nitric acid obtained from the nitrogen in the bomb, wash water from 10 samples was titrated with NaOH (0.1 M). Means of this correction factor were included in the formula used to determine tissue energy. Results are expressed as kJ g^−1^ dry mass.

### Statistical Analysis

The effects of single and combined stressors were analyzed using permutational multivariate analysis of variance (PERMANOVA) based on Euclidean distance with PRIMER v.6^67,68^, where the terms “Temperature” (two levels—ambient and warming), “Salinity variation” (two levels—pulse and press), “Nutrient status” (two levels—natural and enriched) and “Exposure time” (two levels—1 and 3 months) were included as fixed factors for mortality and chlorophyll *a* biomass (Table 2)*.* For energy content and shell mineralization, since measurements were carried out at the individual level, we added the term “aquarium” as a random factor, with “Exposure time”, “Temperature”, “Salinity variation” and “Nutrient status” nested in “Aquarium” (Tables 3, 4). Due to very low concentrations of chlorophyll *a* in the samples, 11 samples fell below detection levels. These were removed from the statistical analysis for chlorophyll *a* as they provided negative values. Beforehand, we tested the effect of the 80 L water bath and mixing tanks on mortality, chlorophyll *a* biomass, energy content and shell mineralization to verify any potential experimental bias. No significant variation was observed. We set the possibility of making a type I error to α = 0.05. Homoscedasticity of residuals was verified using the test of homogeneity of dispersions (PERMDISP). We used pair-wise comparisons among given sources of variation of interest to further evaluate significant interactions to understand which levels differed.

### Interactions interpretation

To help interpret the nature of observed interactions, we used Piggott et al.^14^ interaction classifications based on an additive (A + B) and multiplicative ((A + B) – (A × B)) effects model. The model combines response magnitude and direction (positive or negative) of the combined effects of stressors considered relative to the effect observed under control conditions, as well as the deviation from a null model (additive or multiplicative model) to determine interaction type (synergism and antagonism). Here, we did not have a control condition but compared the combined effect of stressors to a least-stressful condition (ambient temperature, freshwater pulses, no nutrient enrichment). Therefore, when there were no significant interactions between the stressors (i.e. no significant interactions in PERMANOVA), we could identify dominance (i.e. combined effects are equal to that of one of the individual stressors) or additive (i.e. combined effects are equal to the algebraic sum of the individual effects of each stressor) effects^7, 14^. When we identified two- or three-way interaction terms (i.e. significant interaction term in PERMANOVA), we characterized interactions as antagonistic or synergistic and noted their direction (less or more positive or negative than the null model)^15^. We used an additive null model for chlorophyll *a*, shell mineralization and energy content. For mortality, we used a multiplicative null model to correct for the issue that individuals killed by one stressor cannot be killed by another and the model sets the combined mortality to a maximum of 100%^7,69^. We did not consider the source of variation “Exposure time” as a stressor, but when an interaction between a stressor and “Exposure time” was detected, we concluded a dominance effect and verified whether it was consistent over time or not. When only two stressors interacted, we evaluated the results against a null model to detect synergistic or antagonistic interactions and evaluated consistency over time if “Exposure time” also interacted with the response.

## Results

### Microphytobenthos biomass

**Figure 1:**
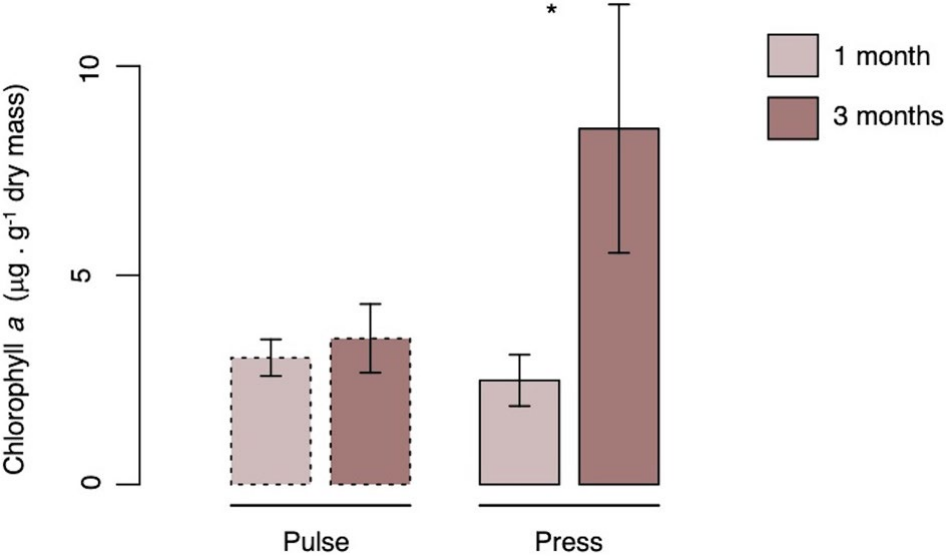
Effect of salinity variation through time on microphytobenthic standing stock. Average chlorophyll *a* concentration (μg g^−1^ dry mass) for salinity variation following one and three months of exposure. Pair-wise contrasts showed a significant difference between one and three-months of exposure within for freshwater presses (p < 0.05) as indicated by the asterisk. Histograms represent mean values, and error bars correspond to standard errors.

In general, chlorophyll *a* concentration in the upper sediment layer was less than 5 μg g^−1^ dry sediment across all treatments but increased when affected by freshwater presses over time, as indicated by the significant Exposure time × Salinity interaction (Fig. 1, Table 2). This demonstrates a dominance of freshwater presses through time, as indicated by the significantly greater chlorophyll *a* concentration after three months exposure to presses and that chlorophyll *a* concentration did not vary over time under the effect of freshwater pulses. Mean concentrations of chlorophyll *a* for all treatments are summarized in Supplementary Table S2.

**Table 2:** Results of PERMANOVA analyses investigating the effect of exposure time (T), temperature (W), salinity variation (S) and nutrient enrichment (N) on mortality levels in the common mussel, *Mytilus* sp. and the Baltic clam, *L. balthica* and on chlorophyll *a* concentration in sediments.

Source of variation	DF	Mortality						Chlorophyll *a*			
		*Mytilus* sp.			*Limecola balthica*			DF	MS	Pseudo F	*P*
		MS	Pseudo F	*P*	MS	Pseudo F	*P*				
T	1	1.065	2.042 × 10^–2^	ns	200.84	0.580	ns	1	160.24	5.340	*
W	1	30.725	0.589	ns	538.95	1.556	ns	1	1.497	4.988 × 10^–2^	ns
S	1	169.93	3.257	ns	181.81	0.525	ns	1	64.46	2.148	ns
N	1	22.834	0.438	ns	132.65	0.383	ns	1	18.869	0.629	ns
T × W	1	9.588	0.184	ns	1.315 × 10^–2^	3.797 × 10^–5^	ns	1	64.366	2.145	ns
T × S	1	1.065	2.042 × 10^–2^	ns	1223.5	3.532	ns	1	120.71	4.023	*
T × N	1	14.323	0.275	ns	104.18	0.301	ns	1	2.134 × 10^–2^	7.112 × 10^–4^	ns
W × S	1	23.57	0.452	ns	15.332	4.426 × 10^–2^	ns	1	20.396	0.680	ns
W × N	1	79.336	1.521	ns	1. 89.85	0.548	ns	1	8.044	0.268	ns
S × N	1	230.33	4.415	*	0.812	2.344 × 10^–3^	ns	1	10.328	0.344	ns
T × W × S	1	9.588	0.184	ns	24.969	7.208 × 10^–2^	ns	1	72.316	2.410	ns
T × W × N	1	0.118	2.269 × 10^–3^	ns	63.66	0.184	ns	1	16.243	0.541	ns
T × S × N	1	14.323	0.275	ns	148.98	0.430	ns	1	5.404	0.180	ns
W × S × N	1	7.367	0.141	ns	61.586	0.178	ns	1	1.893	6.307 × 10^–2^	ns
T × W × S × N	1	0.118	2.269 × 10^–3^	ns	70.089	0.202	ns	1	4.454	0.148	ns
Residual	62	52.175			346.4			50	30.008		
Total	77							66			

The factors temperature (W), salinity variation (S) and nutrient enrichment (N) were set as fixed. Details are provided for degree of freedom (DF), mean square (MS), Pseudo-F and probability level (*P*).

### Mortality and growth

**Figure 2:**
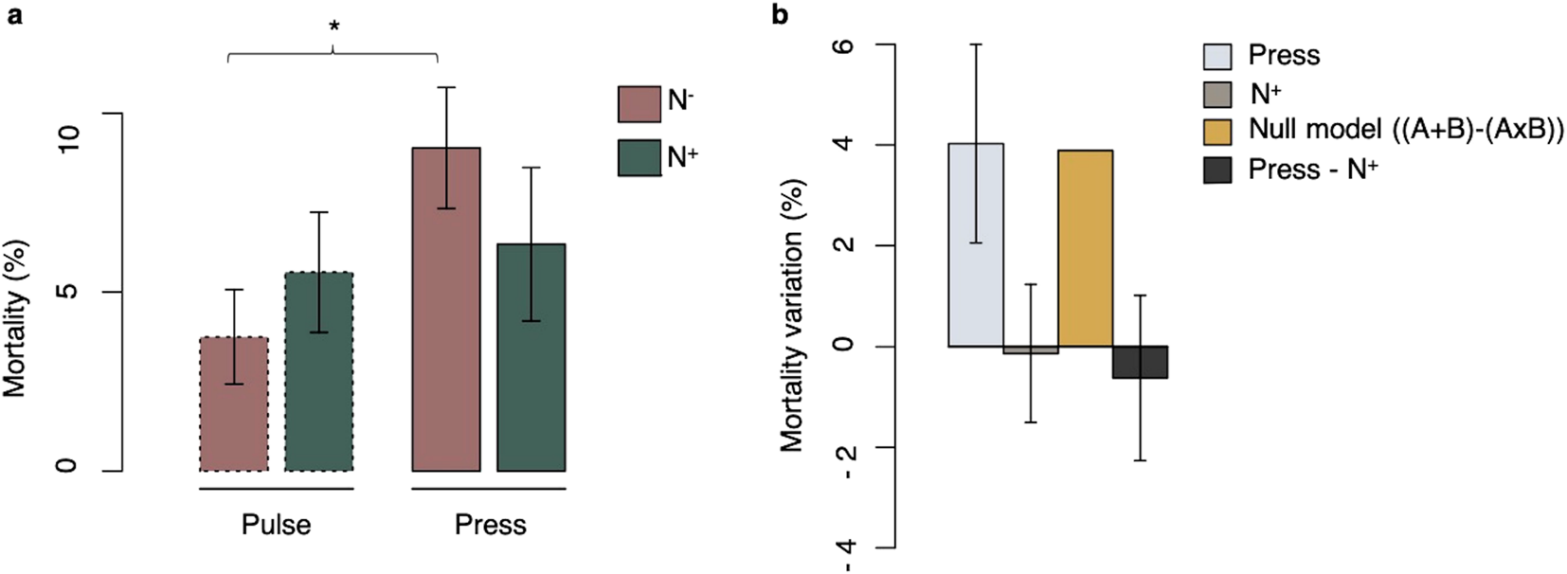
Effect of the interaction between salinity variation and nutrient enrichment on mortality levels in *Mytilus* sp. (**a**) Average mortality level (%) for salinity treatments (S) and nutrient concentrations (N) in *Mytilus* sp. Pair-wise contrasts showed a significant difference between pulses and freshwater presses with natural nutrient concentrations (p < 0.05) as identified by the asterisk. Histograms represent the mean value, and error bars correspond to standard errors. (**b**) Positive antagonistic interaction between freshwater presses and nutrient enrichment based on a null multiplicative model (yellow) ((S + N) − (S × N)). Single effects of stressors, compared to the least stressful conditions, are shown in light blue and gray and a combined effect is shown in dark gray. Error bars correspond to standard errors.

In general, mortality was relatively low across all treatments for *Mytilus* sp., ranging between 0 and 10%, and higher in *L. balthica*, ranging between 10 and 30%. Mortality of *Mytilus* sp. varied significantly as a function of the Nutrient × Salinity interaction (Fig. 2, Table 2). Specifically, mortality in animals exposed to freshwater presses was higher in the absence of nutriment enrichment relative to those with nutriment enrichment. Animals exposed to pulses of freshwater were not affected by nutriment enrichment (Fig. 2a, Table 2). Following the null multiplicative model^7^, this indicates a positive antagonistic interaction (i.e. less positive than the null model) between the press freshwater regime and nutrient enrichment (Fig. 2b). Indeed, examination of individual stressors suggests that the press freshwater regime increased the average mortality by 4% relative to the least stressful conditions. Nutrient enrichment alone had no, or very slight, effects on mortality levels relative to the least stressful conditions. However, the combined effect of a press regime and nutrient enrichment was lower than the multiplicative expectation, thus resulting in reduced mortality levels relative to the least stressful conditions.

*L. balthica* mortality levels were not significantly affected by stressors, individually or in combination. Mean mortality levels for all treatments are summarized for both species in Supplementary Table S2. Finally, no growth was detected for either species across all treatments tested during the exposure period.

### Shell mineralization

No loss of material from empty shells was detected over the experimental period, indicating that passive dissolution of shells did not occur under any experimental conditions tested over the one- and three-month exposure periods. Mean concentrations of all cations measured in both species for all treatments are summarized in Supplementary Table S1.

For *Mytilus* sp., exposure time had a significant effect on Mg^2+^/Ca^2+^ (Table 3) as the ratio was lower following three months than one month of exposure. No other major mineral shell components (i.e. [Ca^2+^], [Mg^2+^], [Sr^2+^] and Sr^2+^/Ca^2+^) varied as a function of any of the evaluated factors.

**Figure 3:**
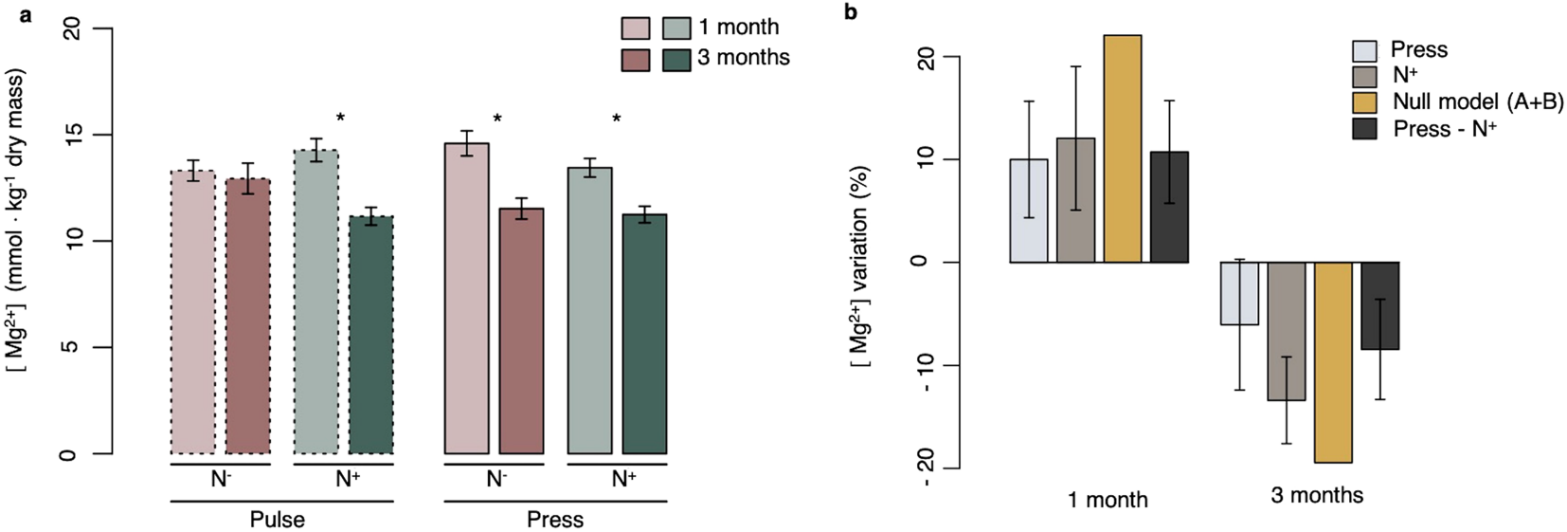
Effect of the interaction between salinity variation and nutrient enrichment through time on magnesium content in the shell of *L. balthica*. (**a**) Average magnesium content (mmol of Mg^2+^ kg^−1^ dry mass) for salinity treatments (S) and nutrient concentrations (N) at one and three months of exposure in *L. balthica*. Pair-wise contrasts showed a significant difference between the two exposure times for different combinations of salinity variation and nutrient conditions (p < 0.05) as indicated by asterisks. Histograms represent mean values, and error bars correspond to standard errors. (**b**) Positive and negative antagonistic interaction between freshwater presses and nutrient enrichment through time based on a null additive model (yellow) (S + N). Single effects of stressors, compared to the least stressful conditions, are shown in light blue and gray and combined effect is shown in dark gray. Error bars correspond to standard errors.

In contrast, several major mineral shell components varied as a function of Time for *L. balthica* (Fig. 3a, Table 3). For example, shell [Mg^2+^] varied as a function of the Exposure time × Salinity × Nutrient interaction such that it was consistently lower following three months incubation in the press treatments, with or without nutrient enrichment—respectively 21% and 16% lower at three and one month, but under freshwater pulses, shell [Mg^2+^] content did not vary without nutrient enrichment but resulted in 22% lower content with increased nutrients following three months incubation. The same pattern was observed for the Mg^2+^/Ca^2+^. Within the pulse treatment, the ratio was lower at three months with increased nutrients and, for the press treatment, it was always lower at three months regardless of nutrient concentrations. Based on a null additive model, the combined stressors consistently resulted in an antagonistic interaction at one and three months but in opposing directions (Fig. 3b). At one-month, individual stressors both had a positive effect, increasing shell [Mg^2+^] when compared to the least stressful conditions. The response to the combined stressors was lower than expected and was classified as a positive antagonistic interaction. In contrast, after three months, individual stressors had reduced shell [Mg^2+^]. The combined effect of the press regime and nutrient enrichment was less negative than the additive expectation, resulting in a negative antagonistic effect. Exposure time alone impacted [Sr^2+^] and Sr^2+^/Ca^2+^ with both being lower following three months (respectively, 19.626 mmol kg^−1^ dry shell and 0.0021) than one-month (respectively, 20.378 mmol kg^−1^ dry shell and 0.0022) exposure. Shell [Ca^2+^] did not vary as a function of any of the combinations of stressors.

**Table 3:** Results of PERMANOVA analyses investigating the effect of exposure time (T), temperature (W), salinity variation (S) and nutrient enrichment (N) on the concentrations of bivalent cations (calcium—Ca^2+^, magnesium—Mg^2+^, strontium—Sr^2+^) (mmol kg^−1^ of dry shell) and their ratios (Ca^2+^/Mg^2+^ and Sr^2+^/Ca^2+^) in the shell of *Mytilus* sp. and *L. balthica*.

	Source of variation	DF	Ca^2+^			Mg^2+^			Sr^2+^			Mg^2+^/Ca^2+^			Sr^2+^/Ca^2+^		
			MS	Pseudo F	*P*	MS	Pseudo F	*P*	MS	Pseudo F	*P*	MS	Pseudo F	*P*	MS	Pseudo F	*P*
*Mytilus* sp.	T	1	1.507 × 10^6^	1.889	ns	101.53	3.113	ns	8.973	0.999	ns	2.070 × 10^–6^	8.443	**	8.964 × 10^17^	1.043	ns
	W	1	2.097 × 10^6^	2.627	ns	100.81	3.091	ns	7.707	0.191	ns	4.172 × 10^–7^	1.704	ns	3.067 × 10^17^	0.358	ns
	S	1	2.178 × 10^5^	0.274	ns	34.584	1.062	ns	0.201	2.406 × 10^–2^	ns	6.561 × 10^–7^	2.679	ns	1.182 × 10^18^	1.374	ns
	N	1	1.651 × 10^5^	0.208	ns	5.999	0.186	ns	14.493	1.612	ns	1.763 × 10^–8^	7.475 × 10^–2^	ns	1.329 × 10^17^	0.157	ns
	T × W	1	5131.9	7.960 × 10^–3^	ns	11.172	0.345	ns	23.721	2.637	ns	7.511 × 10^–8^	0.309	ns	1.166 × 10^18^	1.354	ns
	T × S	1	13,715	1.871 × 10^–2^	ns	36.92	1.133	ns	0.550	6.279 × 10^–2^	ns	4.368 × 10^–7^	1.784	ns	1.678 × 10^17^	0.197	ns
	T × N	1	3.180 × 10^5^	0.400	ns	9.900	0.306	ns	8.775	0.977	ns	3.496 × 10^–8^	0.145	ns	3,471 × 10^17^	0.405	ns
	W × S	1	4.332 × 10^5^	0.544	ns	33.187	1.019	ns	0.143	1.754 × 10^–2^	ns	6.659 × 10^–7^	2.718	ns	3.801 × 10^17^	0.444	ns
	W × N	1	2.778 × 10^5^	0.350	ns	3.926	0.123	ns	1.800	0.201	ns	1.574 × 10^–9^	9.293 × 10^–3^	ns	3.801 × 10^17^	0.444	ns
	S × N	1	1.980 × 10^5^	0.250	ns	0.797	2.670 × 10^–2^	ns	0.253	2.982 × 10^–2^	ns	7.746 × 10^–9^	3.446 × 10^–2^	ns	2.928 × 10^17^	0.342	ns
	T × W × S	1	1.201 × 10^5^	0.152	ns	14.646	0.451	ns	0.472	5.417 × 10^–2^	ns	1.940 × 10^–7^	0.794	ns	4.663 × 10^17^	0.543	ns
	T × W × N	1	1.020 × 10^6^	1.279	ns	70.639	2.167	ns	5.702	0.635	ns	2.972 × 10^–7^	1.215	ns	1.376 × 10^17^	0.162	ns
	T × S × N	1	1.598 × 10^6^	0.202	ns	47.969	1.472	ns	21.879	2.433	ns	4.394 × 10^–7^	1.794	ns	8.225 × 10^17^	0.957	ns
	W × S × N	1	958.03	2.734 × 10^–3^	ns	30.49	0.936	ns	1.372	0.154	ns	3.675 × 10^–7^	1.501	ns	2.862 × 10^17^	0.335	ns
	T × W × S × N	1	5.997 × 10^5^	0.752	ns	27.639	0.849	ns	10.814	1.203	ns	1.207 × 10^–7^	0.495	ns	7.033 × 10^17^	0.818	ns
	A (T × W × S × N)	62	7.960 × 10^5^	2.206	***	32.528	1.490	*	8.970	1.996	***	2.444 × 10^–7^	1.178	ns	8.578 × 10^17^	1.925	***
	Residual	162	3.608 × 10^5^			21.828			4.494			2.076 × 10^–7^			4.457 × 10^17^		
	Total	239															
*Limecola balthica*	T	1	2.868 × 10^5^	0.206	ns	287.58	27.063	****	35.199	4.128	*	3.091 × 10^–6^	28.746	****	2.631 × 10^–7^	9.894	**
	W	1	47.581	3.422 × 10^–5^	ns	9.444 × 10^–2^	8.887 × 10^–3^	ns	0.123	1.446 × 10^–2^	ns	8.226 × 10^–10^	7.650 × 10^–3^	ns	7.470 × 10^–10^	8.809 × 10^–2^	ns
	S	1	33,551	2.413 × 10^–2^	ns	3.051	0.287	ns	0.656	7.693 × 10^–2^	ns	2.145 × 10^–8^	0.199	ns	3.901 × 10^–9^	0.147	ns
	N	1	2.400 × 10^6^	1.726	ns	21.563	2.029	ns	29.957	3.161	ns	7.438 × 10^–8^	0.692	ns	4.013 × 10^–8^	1.509	ns
	T × W	1	853.58	6.138 × 10^–4^	ns	17.612	1.657	ns	0.383	4.492 × 10^–2^	ns	2.422 × 10^–7^	2.252	ns	2.666 × 10^–9^	0.100	ns
	T × S	1	1.173 × 10^6^	0.843	ns	12.966	1.220	ns	1.075	0.126	ns	4.892 × 10^–8^	0.455	ns	1.176 × 10^–8^	0.442	ns
	T × N	1	1.138 × 10^6^	0.818	ns	13.914	1.309	ns	10.186	1.195	ns	7.343 × 10^–8^	0.683	ns	9.009 × 10^–9^	0.339	ns
	W × S	1	1112.8	8.002 × 10^–4^	ns	9.158	0.862	ns	1.159	0.136	ns	1.156 × 10^–7^	1.075	ns	2.068 × 10^–8^	0.778	ns
	W × N	1	5291	3.805 × 10^–4^	ns	12.34	1.161	ns	0.366	4.297 × 10^–2^	ns	1.455 × 10^–7^	1.354	ns	3.636 × 10^–9^	0.137	ns
	S × N	1	24,739	1.780 × 10^–2^	ns	1.464	0.138	ns	1.549	0.182	ns	3.246 × 10^–8^	0.302	ns	1.208 × 10^–8^	0.454	ns
	T × W × S	1	1.893 × 10^6^	1.361	ns	0.514	4.840 × 10^–2^	ns	13.257	1.555	ns	1.302 × 10^–8^	0.121	ns	3.812 × 10^–9^	0.143	ns
	T × W × N	1	1.592 × 10^6^	1.145	ns	13.568	1.277	ns	7.260	0.851	ns	5.059 × 10^–8^	0.471	ns	1.903 × 10^–10^	7.157 × 10^–3^	ns
	T × S × N	1	3.091 × 10^5^	0.222	ns	45.942	4.323	*	0.485	5.692 × 10^–2^	ns	4.093 × 10^–7^	3.806	ns	3.607 × 10^–8^	1.356	ns
	W × S × N	1	7.861 × 10^5^	0.565	ns	0.812	7.641 × 10^–2^	ns	3.726	0.437	ns	1.169 × 10^–9^	1.087 × 10^–2^	ns	1.905 × 10^–10^	7.163 × 10^–3^	ns
	T × W × S × N	1	1.311 × 10^6^	0.943	ns	2.860	0.269	ns	23.984	2.813	ns	1.340 × 10^–7^	1.246	ns	8.152 × 10^–8^	3.065	ns
	A (T × W × S × N)	62	1.395 × 10^6^	2.660	****	10.642	1.454	*	8.540	1.476	*	1.076 × 10^–7^	1.275	ns	2.656 × 10^–8^	0.766	ns
	Residual	161	5.244 × 10^5^			7.320			5.787			8.442 × 10^–8^			3.469 × 10^–8^		
	Total	238															

The factors temperature (W), salinity variation (S), nutrient enrichment (N) were set as fixed, and aquarium (A) as random. Details are provided for degree of freedom (DF), mean square (MS), Pseudo F and probability level (*P*).

### Energy content

In general, *Mytilus* sp. tissue energy content was significantly lower following one-month than three month’s exposure (respectively, 29.634 and 38.550 kJ g^−1^ dry mass). Energy levels were not affected by any single or combined stressors (Table 4).

**Table 4:** Results of PERMANOVA analyses investigating the effect of exposure time (T), temperature (W), salinity variation (S) and nutrient enrichment (N) on energy content in the tissues of *Mytilus* sp. and *L. balthica* (kJ g^−1^ of dry mass).

Source of variation	Energy content							
	*Mytilus* sp.				*Limecola balthica*			
	DF	MS	Pseudo F	*P*	DF	MS	Pseudo F	*P*
T	1	3087.8	18.555	***	1	2188	1.444	ns
W	1	19,818	1.191	ns	1	904.62	0.597	ns
S	1	33.913	0.204	ns	1	39.933	2.635 × 10^–2^	ns
N	1	410.7	2.468	ns	1	2432	1.605	ns
T × W	1	200.8	1.207	ns	1	3372.5	2.225	ns
T × S	1	334.78	2.012	ns	1	153.1	0.761	ns
T × N	1	147.95	0.889	ns	1	7658.5	5.053	*
W × S	1	246.12	1.479	ns	1	790.8	0.528	ns
W × N	1	50.476	0.303	ns	1	2.424	1.599 × 10^–3^	ns
S × N	1	27.132	0.163	ns	1	1846.1	1.218	ns
T × W × S	1	36.692	0.220	ns	1	161.6	0.107	ns
T × W × N	1	23.368	0.140	ns	1	3332.7	2.199	ns
T × S × N	1	6.159	3.701 × 10^–2^	ns	1	4142.8	2.734	ns
W × S × N	1	36.481	0.219	ns	1	4952.7	3.268	ns
T × W × S × N	1	155.6	0.935	ns	1	43.869	2.895 × 10^–2^	ns
A (T × W × S × N)	63	167.96	1.203	ns	62	1546.1	1.462	ns
Residual	81	139.52			81	1057.4		
Total	159				158			

The factors temperature (W), salinity variation (S), nutrient enrichment (N) were set as fixed, and aquarium (A) as random. Details are provided for degree of freedom (DF), mean square (MS), Pseudo F and probability level (*P*).

**Figure 4:**
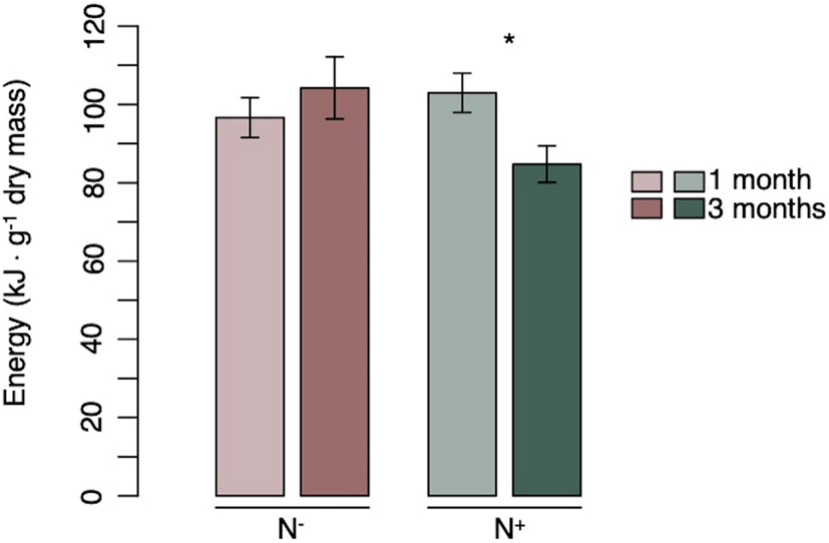
Effect of nutrient enrichment through time on energy content in the tissues of *L. balthica*. Average energy content (kJ g^−1^ dry mass) for nutrient conditions following one and three months exposure. Pair-wise contrasts showed a significant difference between one and three months of exposure under enriched nutrient conditions (p < 0.05) as indicated by the asterisk. Histograms represent mean values, and error bars correspond to standard errors.

Overall, tissue energy content was relatively higher in *L. balthica* than *Mytilus* sp., with values reaching 99.792 and 94.604 kJ g^−1^ dry mass at one and three months, respectively. *L. balthica* tissue energy content was negatively affected by increased nutrients through time, as illustrated by a significant Exposure time × Nutrient interaction (Fig. 4, Table 4). In the absence of enriched nutrients, no significant difference was found between the two exposure times. In contrast, in the presence of added nutrients, there was a significant decrease of energy in tissues after three months of exposition. This highlights the dominance of the factor “Nutrient status” through time, this stressor having a positive effect on the shorter term than following a longer exposure. Mean energy content for all treatments are summarized for both species in Supplementary Table S2.

## Discussion

Identifying and characterizing interactions among multiple stressors is of paramount importance to understand their effects on ecological systems and have been widely declared a major issue for conservation and management^7^. Our work highlights the importance of using appropriately designed experiments to understand the effects of multiple stressors, and does so for numerous responses, providing usable knowledge for management actions^7^. Below, we focus our discussion on the dominance of single stressors, the prevalence of antagonistic interactions between the evaluated stressors, and the relevance of considering results from experimental studies to guide management actions.

### Dominances of single stressors and antagonistic effects

While stressors may interact to create non-linear responses, single stressors may also have dominant effects. Although dominances can be simpler to interpret than other processes, it may also create ecological surprise as stressors may have unexpected effects. For instance, this study shows a dominance of freshwater presses through time on microphytobenthos biomass and of nutrient enrichment on energy content in *L. balthica*. Increased chlorophyll *a* concentration after three months under freshwater presses may be explained by species and assemblages of algae showing marked preferences or tolerances for low salinity conditions^70^. This suggests that salinity is an environmental factor that affects microphytobenthic species composition^70^. Originally, we expected a dominance of nutrient enrichment as field observations and laboratory experiments have shown this factor to stimulate the increase in microphytobenthic biomass^71^. However, nutrients did not increase chlorophyll *a* concentration in the present study. The decrease in chlorophyll *a* may also be interpreted at the community level, although our experiment was not specifically designed to investigate responses at this level of complexity. Increased chlorophyll *a* concentration could be explained by decreased consumption by *L. balthica* in response to stress if the animal reduces deposit-feeding activities^72^. Increased chlorophyll *a* concentration under these conditions may also be explained by decreased consumption by *Mytilus* sp., given that mortality was increased when mussels were subjected to freshwater presses. Finally, as bivalves may discriminate among microalgal species^73^, variation in microphytobenthic species assemblages may have led to selective feeding and thus decreased filtering and deposit-feeding in both *Mytilus* sp. and *L. balthica*^74^.

Interestingly, a similar nutrient enrichment effect pattern over three months exposure time was observed for tissue energy content in *L. balthica*, which was lower in animals exposed to higher concentrations of nutrients at three months. This may be due to increased energetic costs that individuals incur to sustain homeostasis when subjected to toxic effects from different forms of nitrogen (e.g. ammonia)^75^, likely formed during our experiment, but these were not measured in this study. These effects may include proliferation of gill tissue, progressive acidosis, uncoupling of oxidative phosphorylation, and of osmo-ionic disruption^75^.

While synergies are quite easy to understand, antagonisms are less intuitive as it is hard to imagine how two stressors that, individually have negative effects on a given response, together cause a lesser negative effect^7^. An antagonism will more likely indicate that a positive effect of one stressor will overcompensate for the negative effect of another^13^. This type of interaction has been defined in different ways; we propose using the term antagonism as a synonym of “less-than” a predicted null model^14,15^. In our study, this is first illustrated by an antagonistic interaction between freshwater presses and nutrient enrichment for *Mytilus* sp. mortality. Freshwater presses increased mortality by 5% relative to the least stressful condition, while nutrients had no significant effect. Individual effects of nutrient inputs and salinity are well understood in this species and thus interpretation of the observed antagonism may be possible. *Mytilus* sp. is an osmoconformer, such that it maintains its extracellular fluid isotonic relative to the external environment^76^. The species is physiologically unable to maintain its hemolymph osmolarity over a range of salinities^77^ but it may tolerate reduced salinity by using intracellular volume control mechanisms to allow it to continue to feed, respire and maintain general cellular function^77,78^. Many studies have shown the effect of permanent low salinities (generally around 10 psu), frequency, and amplitude of salinity changes on mussel filtration, growth rate, early development and survival^79–82^. Previous studies have found variable effects of nutrient enrichment on bivalves. Positive effects of nutrient input on bivalves, specifically on *Mytilus* sp., include increased assimilation efficiency, biomass, and abundance due to increased food quantity^83,84^. In contrast, negative effects include increased mortality and reduced biomass^85^. This evidence supports the idea that nutrient enrichment has a detrimental or no effect on *Mytilus* sp. under lower osmotic stress (freshwater pulses) but appears to positively affect *Mytilus* sp. under higher osmotic stress (freshwater presses). This positive effect of nutrients under higher osmotic stress on *Mytilus* sp. survival could also have repercussions at the community-level. For instance, considering that food input was not limiting in our study, nutrients may have indirectly altered energy content in *L. balthica* by lowering mortality and increasing assimilation efficiency in *Mytilus* sp., decreasing food deposition to the sediments and availability for *L. balthica*. In fact, it has been shown that *L. balthica* switches between suspension- and deposit-feeding in response to the availability of suspended food particles and that deposit feeding increases with decreasing food concentrations in the water column^86^. Increased deposit feeding could explain why, in contrast to what we had first hypothesized, microphytobenthos was not more abundant under nutrient enriched conditions. This stresses the importance of considering different levels of organization when investigating the effect of stressor interactions, as the presence of species may alter predicted effects.

An antagonistic interaction between salinity variation and nutrient enrichment was also detected for magnesium shell content of *L. balthica*. Magnesium content and [Mg^2+^]/[Ca^2+^] in *L. balthica* were positively affected by both freshwater presses and nutrient enrichment, when compared to the least stressful conditions, following one-month exposure. Their combined effect was smaller than expected, resulting in a positive antagonism. In contrast, longer exposure yielded the opposite situation, with both stressors individually reducing shell magnesium content. Their combined effect was not as great as the null model, creating negative antagonism. This could be explained by several mechanisms. In the short term, individuals may either (1) not be affected by the stressors and be able to invest in mineralization, or (2) increase mineralization efforts in response to stress^87,88^. In contrast, following three-months exposure, these parameters are negatively impacted by the same stressors, with mean values being significantly lower. This may be explained by non-mutually exclusive mechanisms: (1) passive shell dissolution due to fluctuations in carbonate concentrations and seawater pH, (2) active uptake of carbonate ions to buffer fluctuations in extra-cellular fluid osmo-ionic and acid–base status, and (3) changes in active mineralization. We may exclude passive dissolution as shell surface scouring and empty shell mass loss were not observed over the duration of the experiment. However, bivalves and other organisms possessing carbonate skeletons actively dissolve their shell to buffer (during short disturbances) their extra-cellular fluids^89^ to preserve their acid–base status. Acid–base status may be altered by a number of environmental disturbances, including salinity^90^ and nutrient^75^ changes. The modest reduction in [Mg^2+^] observed, confirmed by reduced [Mg^2+^]/[Ca^2+^], could support this pathway of action. Finally, we cannot completely exclude the possibility that the specimens may have also modified their active mineralization.

### Using experimental studies to guide management actions

While identifying the outcome of every possible stressor combination in natural habitats is an impossible task, identifying generalities about stressors and responses through experimental work may create guidelines for conservation and management scientists^7^. For instance, the experimental work we carried out provides useful information for management by (1) informing on which stressors to act upon under different stressor interaction scenarios, and (2) providing details on which responses to investigate in natural ecosystems depending on the management objective.

Antagonistic interactions are often perceived as less of a concern than synergistic ones, since the impact of multiple stressors will be smaller than otherwise predicted, although acting on stressors without considering their potential interaction may waste effort and resources^16^. However, it is important to identify the type of interaction to avoid wrongly direct management efforts and waste resources^16^. Recent meta-analyses of experimental work on multiple stressors in marine, freshwater and terrestrial ecosystems have shown that antagonistic interactions are as common as synergistic interactions^13,91^. When synergistic effects must be managed, acting directly on local stressors will have the greatest ecosystem benefits^16^. In contrast, reducing local antagonistic stressors may have smaller benefits or even worsen stressor impacts^16^. Our results not only inform on stressor interactions, but also suggest that acting upon a stressor, like salinity variation, may result in the greatest benefit for bivalves, in terms of improved survival, and higher energy content in the tissues and magnesium content in the shell. In fact, this stressor had both dominant effects and interacted antagonistically with other stressors. In addition, acting on a local stressor, such as nutrient input, which had a dominant detrimental effect on energy content over a longer period, may be realistic for managers.

Finally, not all evaluated stressors affected all the biological responses of interest for both bivalves and microphytobenthos. This was to be expected as the effects of individual stressors vary with the level of biological complexity^15,28^, affecting some pathways, but not others. This implies that physiological/individual, population or community/ecosystem responses may be associated with more than one type of interaction^7^. For example, population-level responses tend to be most synergistically impacted by multiple stressors^13^. This indicates that focusing exclusively on individual-level responses could grossly underestimate population and ecosystem implications, with consequent implications for management and conservation actions^15^. For instance, our results show that stressors, alone and in combination, impacted individual species responses at both the population (mortality) and individual levels (shell mineralization, energy content). These results may help interpret specific changes in the biology of target species of interest for management and ultimately help understand changes in community responses^15^.

## Conclusion and recommendations

In conclusion, experimental approaches, when based on realistic baseline conditions, are useful to help identify the stressors to act upon to maximize environmental management outcomes; efforts that are usually constrained by time and resources^92^. We also believe that focusing research on relevant local stressors (salinity variation, nutrient enrichment), within the context of global changes, on which managers can act directly, will be more useful for management and conservation actions.

We further believe that purposely designed experimental studies may inform managers on which component of the environment action is necessary. To that end, experiments must be designed to identify pathways of action and consider responses at the cellular, physiological, population and community level^15^. For example, results on mortality and cascading effects will be of great help for conservation practices, where the ultimate goal is often to maximize a species’ demography and biodiversity. On the other hand, energy content results might be of greater interest for managers who deal with maintaining good levels of energy flow in trophic cascade in natural ecosystems, as well as ensuring a good status of commercial species, such as the blue mussel.

Experimental methods can be used to identify synergistic and antagonistic responses of multiple stressors, but will inevitably reach a complexity limit, as the feasibility of experiments decreases with the number of stressors considered. Generalities identified from experiments need to be validated using other methods, such as field observations and surveys, as well as in situ experiments, to take into account environmental complexity and feedbacks. We strongly believe that the integration of different approaches will ultimately contribute to addressing the challenges related to minimizing the cumulative impacts of future and ongoing multiple stressors on marine ecosystems.

## Supplementary Information

Supplementary Information
