# Supplementary material for: Environmental stressors, complex interactions and marine benthic communities’ responses
Source: Sci Rep. 2021 Feb 18;11:4194. doi: 10.1038/s41598-021-83533-1 (PMC7892560; doi:10.1038/s41598-021-83533-1)
Supplement: Supplementary file 1 — Supplementary Information [file 41598_2021_83533_MOESM1_ESM.pdf]

Environmental stressors, complex interactions and marine benthic communities' responses.

Charlotte Carrier-Belleau[1*], David Drolet[2], Christopher W. McKindsey[1,2], Philippe Archambault[1]

[1] Québec-Océan, Department of Biology, Université Laval, Quebec, Canada,1045, av. de la Médecine, Québec G1V 0A6, Canada

[2] Maurice Lamontagne Institute, Fisheries and Oceans Canada, 850 route de la Mer, Mont-Joli G5H 3Z4

***Correspondence author:** charlotte.carrier.belleau@gmail.com

Supplementary file

Supplementary Figure 1: Schematic representation of experimental design. The experimental set up consisted of two head tanks (3 and 27 °C), eight independent mixing tanks (n=2 salinity/temperature^{-1} treatment) and 80 PVC cylinders (experimental units) distributed in 10 water baths (5 per temperature). The desired temperatures were obtained using16 titanium coils immersed in the two head tanks (8 per head tank) to warm or cool filtered sea water of freshwater. We obtained pulses and presses of freshwater using flowmeters and timers. Every mixing cylinder were linked to ten haphazardly selected experimental cylinders that were distributed in the water baths. Water baths were randomly distributed in the room and each of them housed two replicates for each of the treatment (i.e. combinations of salinity and nutrient at a given temperature (8 cylinders per water bath). Nutrient enrichment was obtained using controlled-release pellets that were placed in the sediments, 8 cm from the surface. The cylinders were exposed to a 12-h photoperiod (7:00 – 19:00) by using LED panels (72 WATTS). Half the cylinders in each tank were collected after 1 month and three months of exposure.

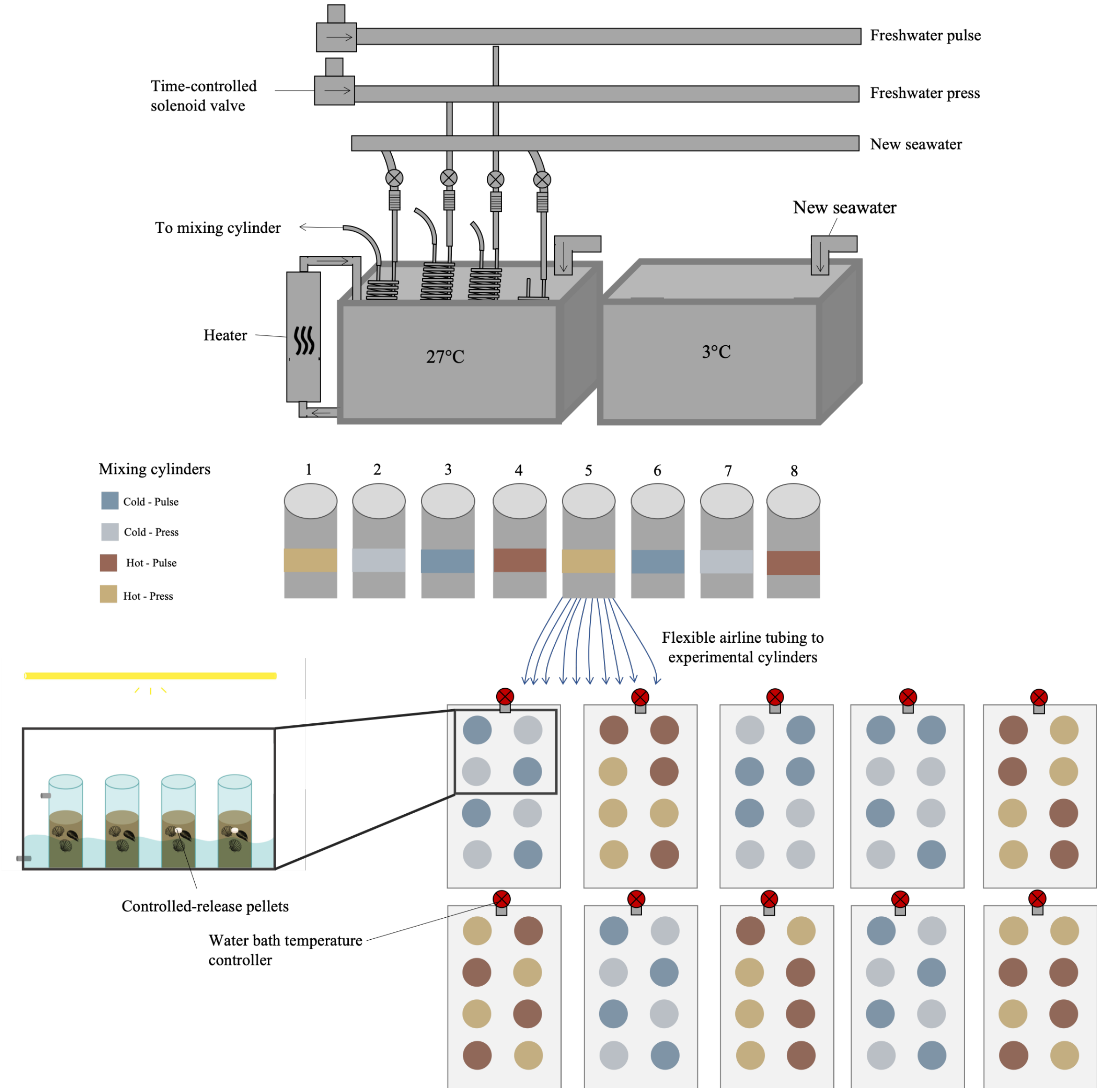

Supplementary Table 1 : Mean concentrations (mmol · Kg^{-1} of dry shell) (± SE) of bivalent cations (calcium – Ca^{2+}, magnesium – Mg^{2+}, manganese – Mn^{2+}, strontium – Sr^{2+}, barium – Ba^{2+} and boron – B^{2+}) in the shell of the common mussel, *Mytilus* sp. and the Baltic clam, *Limecola balthica* for all treatments after a month and three months of exposition to experimental conditions (n= 15 *per* species).

	Treatments				Cation concentration					
	**Exposure**	**Temperature**	**Salinity variation**	**Nutrient**	**Ca^{2+}**	**Mg^{2+}**	**Mn^{2+}**	**Sr^{2+}**	**Ba^{2+}**	**B^{2+}**
Mytilus sp.	1	Ambient	Pulse	N^-	9428.1136 (± 11.4183)	30.3661 (± 1.0505)	0.1320 (± 0.0124)	15.8535 (± 0.7409)	0.0203 (± 0.0019)	1.2024 (± 0.0270)
	1	Ambient	Pulse	N^+	9504.7409 (± 127.6315)	31.3106 (± 1.4314)	0.1497 (± 0.0138)	15.0321 (± 0.3622)	0.0207 (± 0.0012)	1.1476 (± 0.0551)
	1	Ambient	Press	N^-	9444.7689 (± 336.2773)	32.8007 (± 1.7113)	0.1285 (± 0.0191)	15.7429 (± 0.6023)	0.0221 (± 0.0029)	1.2098 (± 0.0584)
	1	Ambient	Press	N^+	9278.0931 (± 285.2551)	32.2888 (± 1.5126)	0.1364 (± 0.0114)	15.0897 (± 0.6812)	0.0238 (± 0.0028)	1.1782 (± 0.0342)
	1	Warming	Pulse	N^-	9367.8147 (± 118.7082)	31.9909 (± 0.8602)	0.1049 (± 0.0116)	17.1776 (± 0.5788)	0.0207 (± 0.0032)	1.1957 (± 0.0445)
	1	Warming	Pulse	N^+	9086.7927 (± 204.4628)	28.2248 (± 1.3005)	0.1415 (± 0.0117)	14.8971 (± 0.5896)	0.0204 (± 0.0018)	1.0442 (± 0.0357)
	1	Warming	Press	N^-	9271.6464 (± 235.1574)	31.1041 (± 1.4963)	0.1117 (± 0.0092)	15.6237 (± 0.6972)	0.0198 (± 0.0020)	1.1420 (± 0.0450)
	1	Warming	Press	N^+	9173.0400 (± 142.7061)	31.7302 (± 1.6350)	0.1355 (± 0.0098)	15.8618 (± 0.5430)	0.0269 (± 0.0025)	1.1651 (± 0.0366)
	3	Ambient	Pulse	N^-	9765.6305 (± 152.8206)	30.2493 (± 1.3709)	0.1388 (± 0.0096)	16.6174 (± 0.6902)	0.0222 (± 0.0024)	1.1932 (± 0.0400)
	3	Ambient	Pulse	N^+	9590.5820 (± 154.6268)	30.0395 (± 1.2443)	0.1123 (± 0.0133)	16.2888 (± 0.7312)	0.0262 (± 0.0023)	1.1781 (± 0.0454)
	3	Ambient	Press	N^-	9543.1837 (± 144.6774)	32.3938 (± 1.1121)	0.1331 (± 0.0115)	16.8805 (± 0.5514)	0.0279 (± 0.0018)	1.1204 (± 0.0309)
	3	Ambient	Press	N^+	9377.9148 (± 137.8018)	30.2105 (± 0.9428)	0.0970 (± 0.0103)	16.0179 (± 0.5275)	0.0231 (± 0.0018)	1.1580 (± 0.0385)
	3	Warming	Pulse	N^-	9129.8869 (± 121.8033)	28.4314 (± 1.2964)	0.1234 (± 0.0127)	15.0065 (± 0.7956)	0.0219 (± 0.0023)	1.0642 (± 0.0409)
	3	Warming	Pulse	N^+	9566.8098 (± 144.0950)	30.8660 (± 1.6130)	0.1317 (± 0.0119)	16.2050 (± 0.7187)	0.0226 (± 0.0017)	1.1497 (± 0.0390)
	3	Warming	Press	N^-	9438.5991 (± 159.2855)	28.2015 (± 0.6802)	0.1109 (± 0.0133)	16.0628 (± 0.6011)	0.0257 (± 0.0031)	1.0729 (± 0.0346)

	3	Warming	Press	N$^+$	9406.2468 (± 141.8124)	28.6012 (± 1.2340)	0.1139 (± 0.0214)	15.4619 (± 0.4438)	0.0256 (± 0.0021)	1.1003 (± 0.0267)
L.balthica	1	Ambient	Pulse	N$^-$	9298.3858 (± 209.0913)	12.6084 (± 0.7898)	0.2319 (± 0.0853)	20.6026 (± 0.6720)	0.0519 (± 0.0033)	0.7045 (± 0.0458)
	1	Ambient	Pulse	N$^+$	9475.9505 (± 238.7101)	14.1295 (± 0.8801)	0.1784 (± 0.1060)	20.1513 (± 0.6421)	0.0499 (± 0.0024)	0.7975 (± 0.0599)
	1	Ambient	Press	N$^-$	9326.2758 (± 252.0440)	13.8534 (± 3.5769)	0.2233 (± 0.0611)	20.8490 (± 0.5805)	0.0488 (± 0.0013)	0.7867 (± 0.0182)
	1	Ambient	Press	N$^+$	9379.3672 (± 146.5980)	13.9601 (± 0.6489)	0.2327 (± 0.0761)	20.5077 (± 0.9622)	0.0500 (± 0.0014)	0.7295 (± 0.0435)
	1	Warming	Pulse	N$^-$	9307.3473 (± 184.9988)	14.0154 (± 0.5417)	0.1790 (± 0.0339)	20.7478 (± 0.7478)	0.0501 (± 0.0018)	0.7973 (± 0.0382)
	1	Warming	Pulse	N$^+$	9078.5364 (± 239.1525)	14.3926 (± 0.7783)	0.2222 (± 0.2258)	20.2981 (± 0.6024)	0.0498 (± 0.0021)	0.7134 (± 0.0489)
	1	Warming	Press	N$^-$	9617.6594 (± 305.0899)	15.3342 (± 0.9593)	0.2156 (± 0.1169)	18.4653 (± 0.5796)	0.0535 (± 0.0031)	0.8223 (± 0.0427)
	1	Warming	Press	N$^+$	9381.6205 (± 147.9885)	12.8999 (± 0.5885)	0.2167 (± 0.1221)	19.2735 (± 0.6712)	0.0489 (± 0.0013)	0.8101 (± 0.0360)
	3	Ambient	Pulse	N$^-$	9711.4646 (± 342.6353)	12.9500 (± 1.0176)	0.2283 (± 0.1410)	21.1321 (± 0.7336)	0.0498 (± 0.0024)	0.7939 (± 0.0542)
	3	Ambient	Pulse	N$^+$	8839.4130 (± 182.4537)	11.2147 (± 0.5455)	0.1756 (± 0.0940)	20.1280 (± 0.7458)	0.0410 (± 0.0019)	0.6699 (± 0.0288)
	3	Ambient	Press	N$^-$	9337.2629 (± 293.0210)	12.1657 (± 0.8225)	0.1733 (± 0.0453)	19.9210 (± 0.6019)	0.0478 (± 0.0021)	0.8041 (± 0.0249)
	3	Ambient	Press	N$^+$	9191.9483 (± 142.0216)	11.8556 (± 0.6084)	0.2431 (± 0.1192)	19.9914 (± 0.6133)	0.0458 (± 0.0020)	0.7347 (± 0.0436)
	3	Warming	Pulse	N$^-$	9459.4785 (± 170.6606)	12.9316 (± 1.0490)	0.2048 (± 0.0900)	19.3587 (± 0.7159)	0.0476 (± 0.0019)	0.6750 (± 0.0388)
	3	Warming	Pulse	N$^+$	9480.1413 (± 177.3353)	11.0966 (± 0.5784)	0.2538 (± 0.0873)	20.7180 (± 0.5264)	0.0452 (± 0.0008)	0.7139 (± 0.0399)
	3	Warming	Press	N$^-$	9315.8401 (± 162.2693)	10.8853 (± 0.5224)	0.1889 (± 0.0503)	19.6614 (± 0.3974)	0.0486 (± 0.0017)	0.6748 (± 0.0298)
	3	Warming	Press	N$^+$	8944.1525 (± 176.0021)	10.6791 (± 0.4394)	0.1459 (± 0.0579)	18.5370 (± 0.3754)	0.0413 (± 0.0014)	0.6978 (± 0.0228)

Supplementary Table 2: Mean (± SE) levels of mortality (%) (n=5 *per* species) and energy content (n=10 *per* species) (kJ·g^{-1} of dry mass) in the tissues of the common mussel*, Mytilus* sp. and the Baltic clam, *Limecola balthica* and chlorophyll a concentration (µg·g^{-1} of dry sediment) (n = 3) in the first cm of sediments for all treatments after one month and three months of exposition to experimental conditions.

Treatments				Measured variables				
Exposure	Temperature	Salinity variation	Nutrient	*Mytilus* sp.		*Limecola balthica*		Chlorophyll *a*
				Mortality	Energy	Mortality	Energy	
1	Ambient	Pulse	N^-	5.0 (± 3.1)	26.2752 (± 3.3686)	10.4 (± 7.3)	116.6202 (± 6.2332)	3.3747 (± 1.1806)
1	Ambient	Pulse	N^+	4.7 (± 3.0)	27.2893 (± 3.3621)	12.5 (±4.0)	98.9805 (± 5.7659)	1.9107 (± 0.1371)
1	Ambient	Press	N^-	10.0 (± 4.7)	36.1397 (± 4.3396)	14.2 (± 5.3684)	91.1132 (± 8.4531)	1.0039 (± 0.6786)
1	Ambient	Press	N^+	2.5 (± 2.5)	29.2598 (± 2.2800)	22.5 (± 9.2)	101.7498 (±	0.8303 (± 0.3567)
1	Warming	Pulse	N^-	0.0	28.9262 (± 3.0657)	22.5 (± 10.8)	84.7630 (± 14.4993)	3.5038 (± 1.0)
1	Warming	Pulse	N^+	5.0 (± 3.1)	27.7767 (± 1.5139)	15.0 (± 9.2)	107.9813 (±	3.1786 (± 0.8353)
1	Warming	Press	N^-	10.0 (± 2.5)	29.5295 (± 1.0640)	22.5 (± 10.0)	93.9861 (± 8.2998)	4.8465 (± 1.5413)
1	Warming	Press	N^+	5.0 (± 2.5)	31.9414 (± 1.6470)	20.7 (± 5.2)	103.1399 (± 9.2750)	2.3474 (± 0.4885)
3	Ambient	Pulse	N^-	5.0 (± 3.1)	40.4966 (± 3.7416)	17.5 (± 5.0)	87.4022 (± 14.4399)	3.5336 (± 1.5305)
3	Ambient	Pulse	N^+	4.7 (± 2.9)	35.4112 (± 5.5900)	30.0 (± 8.5)	87.2255 (± 10.6130)	2.3434 (± 0.8373)
3	Ambient	Press	N^-	9.4 (± 6.0)	38.0814 (± 3.9068)	12.5 (± 5.1)	99.3762 (± 17.6456)	4.6927 (± 0.7281)
3	Ambient	Press	N^+	5.0 (± 3.1)	34.5830 (± 2.3424)	12.5 (± 6.0)	77.5575 (± 5.7284)	2.0808 (± 0.5583)
3	Warming	Pulse	N^-	0.0	46.7175 (± 6.0771)	27.5 (± 13.3)	86.9658 (± 6.7733)	2.9478 (± 1.1834)
3	Warming	Pulse	N^+	5.0 (± 3.0619)	37.5943 (± 5.7252)	30.0 (± 14.0312)	94.2176 (± 6.8599)	4.8042 (± 2.4344)
3	Warming	Press	N^-	6.3 (± 3.6084)	41.6656 (± 6.1144)	15.6250 (± 7.8644)	143.1132 (±	5.6307 (± 3.1376)
3	Warming	Press	N^+	5.0 (± 3.0619)	33.8463 (± 2.3262)	20.3571 (± 2.8571)	79.4560 (± 13.5844)	5.1003 (± 0.8855)
